# PrePex circumcision surveillance: Adverse events and analgesia for device removal

**DOI:** 10.1371/journal.pone.0194271

**Published:** 2018-03-26

**Authors:** Limakatso Lebina, Minja Milovanovic, Kennedy Otwombe, Pattamukkil Abraham, Mmatsie Manentsa, Susan Nzenze, Neil Martinson

**Affiliations:** 1 Perinatal HIV Research Unit (PHRU), SA MRC Soweto Matlosana Collaborating Centre for HIV/AIDS and TB, Faculty of Health Sciences, University of the Witwatersrand, Johannesburg, South Africa; 2 Center for TB Research, Johns Hopkins University, Baltimore, MD, United States of America; Cardiff University, UNITED KINGDOM

## Abstract

**Background:**

The PrePex medical male circumcision (MMC) device is relatively easy to place and remove with some training. PrePex has been evaluated in several countries to assess feasibility and acceptability. However, several studies have reported pain associated with removal.

**Objective:**

To assess safety of PrePex and whether analgesia administered prior to removal reduces pain experienced by participants.

**Methods:**

A multi-site non-randomized, prospective cohort study in which adult (18–45 years old) males requesting PrePex device male circumcision, were enrolled in six South African clinics from July 2014 to March 2015. Participants were routinely provided with analgesia shortly after the surveillance commenced following a protocol review. Analgesia regimen for device removal depended on medication availability at clinics.

**Results:**

Of 1023 enrolled participants who had PrePex placed, 98% (1004) had the device removed at a study clinic. Their median age was 25 (IQR: 21–30) years. HIV sero-positivity was 3.6% (37/1023). Nurses placed and removed half of all devices. Adverse events were experienced by 2.4% (25/1023) of participants; 15 required surgical intervention: device displacement (5/14), early removals (3/14), self-removals (5/14) and insufficient skin removed (2/14). Majority (792: 79%) of participants received analgesia. Most received either paracetamol-codeine (33%), lidocaine (29%) or EMLA and Oral Combination (28%). A lower proportion of participants who received any analgesia (except for lidocaine) prior to PrePex removal experienced severe pain compared to those who received no analgesia (16.6% vs. 29%: p = 0.0001).

**Conclusion:**

Reported adverse events during this PrePex active surveillance were similar to previous reports and to those of surgical circumcision. Pain medication provided prior to removal is effective at decreasing severe pain during PrePex device removal.

## Background

There are an estimated 7 million people living with HIV in South Africa; HIV prevalence 19.2% in adults aged 15–49 years [1]. HIV incidence is still unacceptably high, therefore HIV prevention programs remain critical [2]. In the period between 2008 and 2014, over 1.8 million circumcisions were performed in South Africa, 43% of the target to achieve 80% medical circumcision coverage in males [2]. Innovative approaches are required to improve uptake of medical male circumcision (MMC) services [3], especially MMC circumcision devices of which PrePex is one.

The PrePex medical male circumcision (MMC) device has been pre-qualified by the World Health Organization (WHO) as an option for non-surgical circumcision for the expansion of voluntary MMC for HIV prevention [3]; subsequent to recommendations by the WHO that countries with high HIV/AIDS incidence and prevalence should scale-up voluntary MMC as one of the methods for HIV prevention [4] to reduce the risk of acquiring HIV in heterosexual males [5–7].

The PrePex MMC device has been evaluated in many countries for context specific feasibility and acceptability and minimal adverse events were reported [3, 8, 9]. PrePex MMC device has also been found to be cost-effective in multiple studies [10, 11], safe and appropriate for overburdened health systems [12]. A programmatic evaluation of this device showed that pain was experienced by most participants during PrePex removal [13]. However, there is no data on how to improve pain management during removal. The objective of this study therefore was to report safety of PrePex, and whether analgesia (oral or combination of oral and topical) prior to removal reduces pain at removal.

## Methods

This was a non-randomized prospective cohort study in which adult (18–45 years old) males requesting male circumcision and willing to be circumcised using the PrePex device were enrolled from July 2014 to March 2015. The design was based on the WHO Framework for Evaluation of MMC Devices (active surveillance) and follows recommendations provided by the WHO Technical Advisory Group on Innovations in Male Circumcision (TAG)[14]. To be eligible for inclusion, males had to know their HIV status and if HIV positive, CD4 count in the three months prior to circumcision had to be >350 cells/mm^3^. Prospective participants with medical or surgical conditions that were contraindicated for PrePex circumcision or were unwilling to return for the required follow-up visits or were prisoners were excluded from the study. All participants provided written informed consent for inclusion into the study.

### Study setting

The study was conducted in six medical male circumcision clinics, across four provinces in South Africa in: Johannesburg, Soweto, Ekurhuleni, eMalaheleni, Klerksdorp and Bloemfontein.

All six sites provided both surgical (usually forceps guided for older boys and men and dorsal slit for younger boys) and PrePex circumcision [8, 9, 13, 15]. HIV testing and counselling was provided at all the sites for participants who do not know or had no recent record of their HIV status.

### Ethics statement

The Institutional Review Board of the University of the Witwatersrand Ethics Committee approved this study.

### Study procedures

Following written informed consent to participate, all participants answered a baseline questionnaire after a brief directed medical examination that assessed suitability for PrePex circumcision prior to PrePex placement. The data collected on the questionnaire included their age, occupation, HIV status, current dwelling and current treatments. Follow-up questionnaires collected at each follow-up visit focused on duration and complications related to circumcision with PrePex. Follow-up visits were conducted at one week post-placement for removal, day 21-post application for wound review and day 49-post application to assess complete healing. Visits after device removal were either conducted at the clinic or telephonically. In addition, all clients were instructed to return to the clinic for any unscheduled visit if they experienced any adverse events or complications with the device or their circumcision wound.

Manufacturer’s instructions for use of the PrePex device initially did not recommend analgesia. Therefore, at the start of this surveillance study no analgesia was offered to participants before device removal. However, shortly after study start, based on the analysis of prior data [13], a study decision was taken to encourage all sites to provide analgesia routinely prior to removal. As sites had a variety of analgesia in stock they were requested to use whatever they had available, taking into account clinically relevant history. Analgesia therefore included a diverse range of oral and topical agents: EMLA cream (a topical analgesic consisting of 25mg per gram of lidocaine and 25mg per gram of prilocaine), lidocaine spray, ibuprofen tablets, paracetamol tablets, and paracetamol-codeine and ibuprofen-codeine fixed dose combination (FDC) tablets; provided as individual medications or a combination of oral and topical medication. For example, some participants received paracetamol-codeine or ibuprofen orally and EMLA cream applied topically (EMLA and Oral Combination). When participants returned for PrePex removal, most were offered analgesia, 30 minutes to an hour preceding the start of the removal procedure either oral or topical and or a combination at the discretion of the health care worker. We recorded whether or not analgesia was administered and the type/s. In this analysis, medication was grouped as follows: any medication, no medication, EMLA and Oral Combination, ibuprofen (any ibuprofen containing medication), lidocaine and paracetamol-codeine (any paracetamol containing medication).

Outcome measures were pain at the time of removal measured using a visual analogue scale (VAS) to rate pain from 0 (very happy, no hurt), 2 (hurts just a little bit), 4 (hurts a little more), 6 (hurts even more), 8 (hurts a whole lot) to 10 (hurts as much as you can imagine). The five-category pain levels reported by participants during circumcision were categorized into three grades: none-or-mild (0–2 VAS), moderate (4–6 VAS) and severe (8–10 VAS) [8, 13].

All the sites were trained to record adverse events (AEs) according to the PSI/COSECS AE Action Guide for Voluntary Medical Male Circumcision (VMMC) as modified for the PrePex device circumcision [13]. As this surveillance study was part of a MMC program only moderate and severe AEs were reported. Mild adverse events that required no intervention were not reported.

### Statistical analysis

All tests were conducted at the significance level of 0.05. Descriptive statistics (frequency, median, interquartile ranges, percentages) were used to describe quantitative variables. The primary outcomes assessed included: medication type, pain rating during removal for those with medication and those without medication and adverse events. Sstatistical analyses were conducted in Microsoft Excel and SAS Enterprise Guide 7.1 (SAS Institute, Cary NC). The proportion of participants experiencing pain at each level was determined and compared between groups using the Chi-square test of proportions. Those who removed PrePex device prior to day five were considered as early removals and after day eight as late removals.

### Sample size and data collection

The sample size was informed by the WHO Framework for Clinical Evaluation of Devices for Adult Male Circumcision for a cohort field study evaluating safety of procedure and removal, clinical adverse events and device related incidents [14].

Data was collected by the clinical staff members (nurses, doctors and clinical associates) using paper case report forms and then dual entered into an electronic database.

## Results

**Fig 1:**
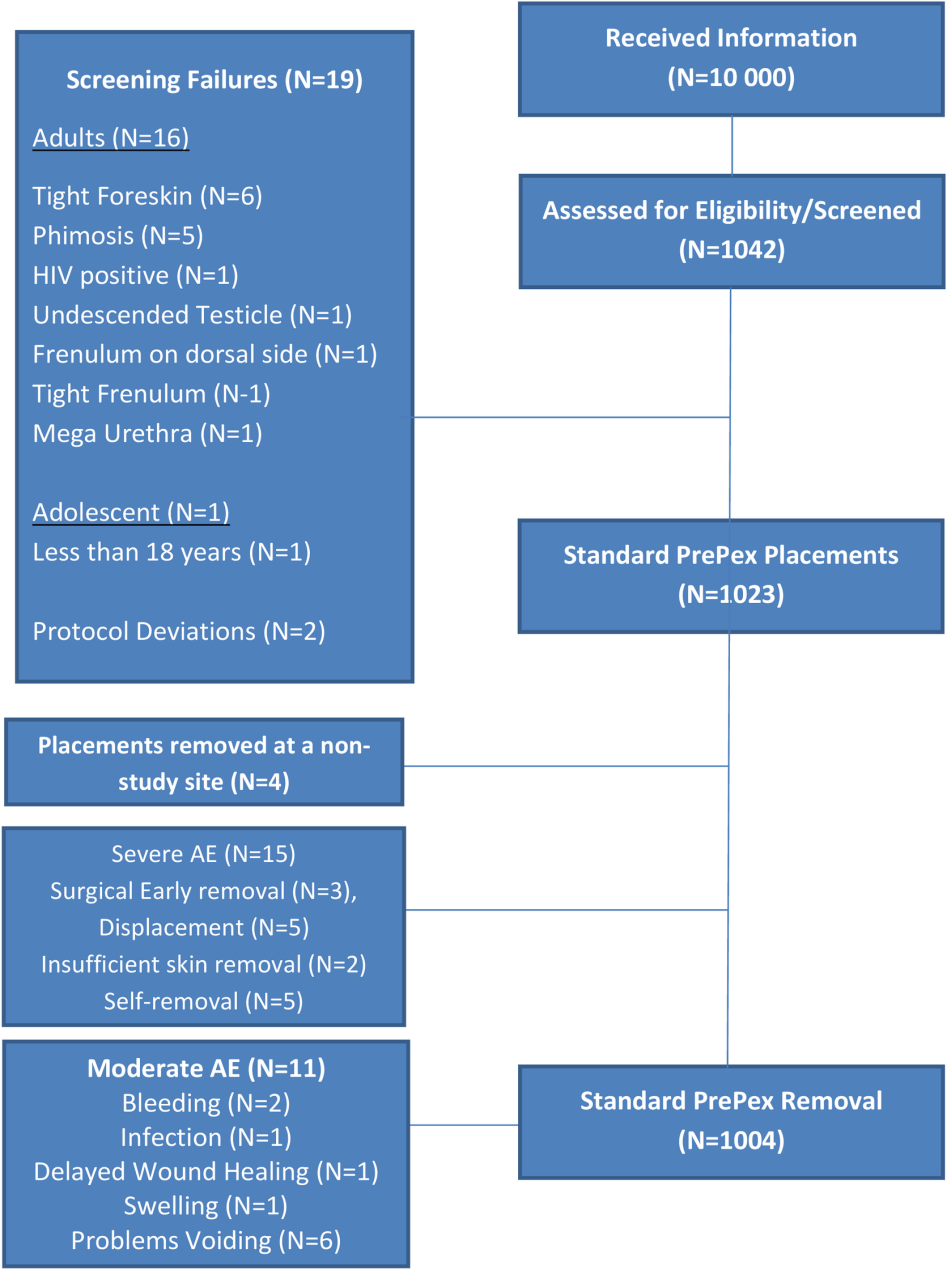
PrePex flowchart.

Approximately 10 000 adult males (aged 18–45 years) received PrePex information across the six sites. A total of 1042 (10%) who expressed interest in PrePex circumcision were assessed for eligibility into the study. Of those, 19/1042 (1.8%) were screening failures including 16 adults, one adolescent (17 years old) and 2 participants were classified as protocol deviations. The commonest reason for adult screening failures was phimosis and tight foreskin (11/16) (Fig 1). A total of 1023 participants were therefore enrolled into the study and received a PrePex placement procedure. Of those, 98% (1004/1023) were removed according to manufacturer guidelines; others were removed too early (0–5 days) or too late (≥9 days) after placement.

The median age of participants was 25 (IQR: 21–30) years and there were no major differences in age across the six sites. Most participants had access to water either inside their yard (41.8%) or inside their dwelling (54.4%) (Table 1). About half of the participants reported being employed (44.5%) during the study, and 7.1% (31/407) were mineworkers, though most (75.9%: 22/29) reported working above ground. Amongst the participants 3.6% (37/1023) were HIV positive with a median CD4 count of 480 (IQR: 392–605) cells/mm^3^.

**Table 1:** Demographics of the study population.

Variables		Number	Percentage
**Age**	Median	25 years	
	IQR	21–30 years	
**Employment Status**	Employed	556/1001	55.5%
	Unemployed	445/1001	44.5%
	Mineworkers	31/438	7.1%
**Residential Area**	Township/Location	853/994	85.8%
	Suburb/Town	120/994	12.1%
	Farm/Village	21/994	2.1%
**Type of Dwelling**	Brick/Concrete House	797/1022	78.0%
	Garage/One Room	62/1022	6.1%
	Shack	155/1022	15.2%
	Other	8/1022	0.8%
**Water**	Piped water inside the dwelling	556/1022	54.4%
	Piped water inside the yard	427/1022	41.8%
	Other	39/1022	3.8%

Overall, the most common PrePex device sizes used were of diameter 28mm (B) and 30mm (C) (61.5%: 615/1019). Nurses did half of the PrePex placements (50.1%: 510/1003) and removals (59.6%: 597/1003) while medical officers placed 21.8% (222/1019) and removed 17.7% (178/1003) of PrePex devices. Majority of participants (88.6%: 881/994) resumed normal activity following the placement of the PrePex device and 11.4% (113/994) reported taking time off from work.

### Adverse events

Amongst the PrePex MMC device participants 2.5% (26/1023) experienced adverse events. Of these, 11 were moderate: bleeding (2/11), infection (1/11), problems voiding (6/11), delayed wound healing (1/11) and swelling (1/11). All moderate adverse event cases were resolved with recommended treatment. There were 15 severe adverse events—requiring additional surgical intervention. These include: displacement (5/15), early removals (3/15), self-removals (5/15) and insufficient skin removals (2/15). Two cases of early removals presented at two different facilities with severe pain on the same day as placement and requested removal of the device. Those that had early removal had surgical circumcision done and were followed up as per the surgical circumcision guidelines. There were no reported serious adverse events that were life threatening or required hospitalization. No participants were diagnosed with Tetanus in this study.

### Analgesia for device removal

**Fig 2:**
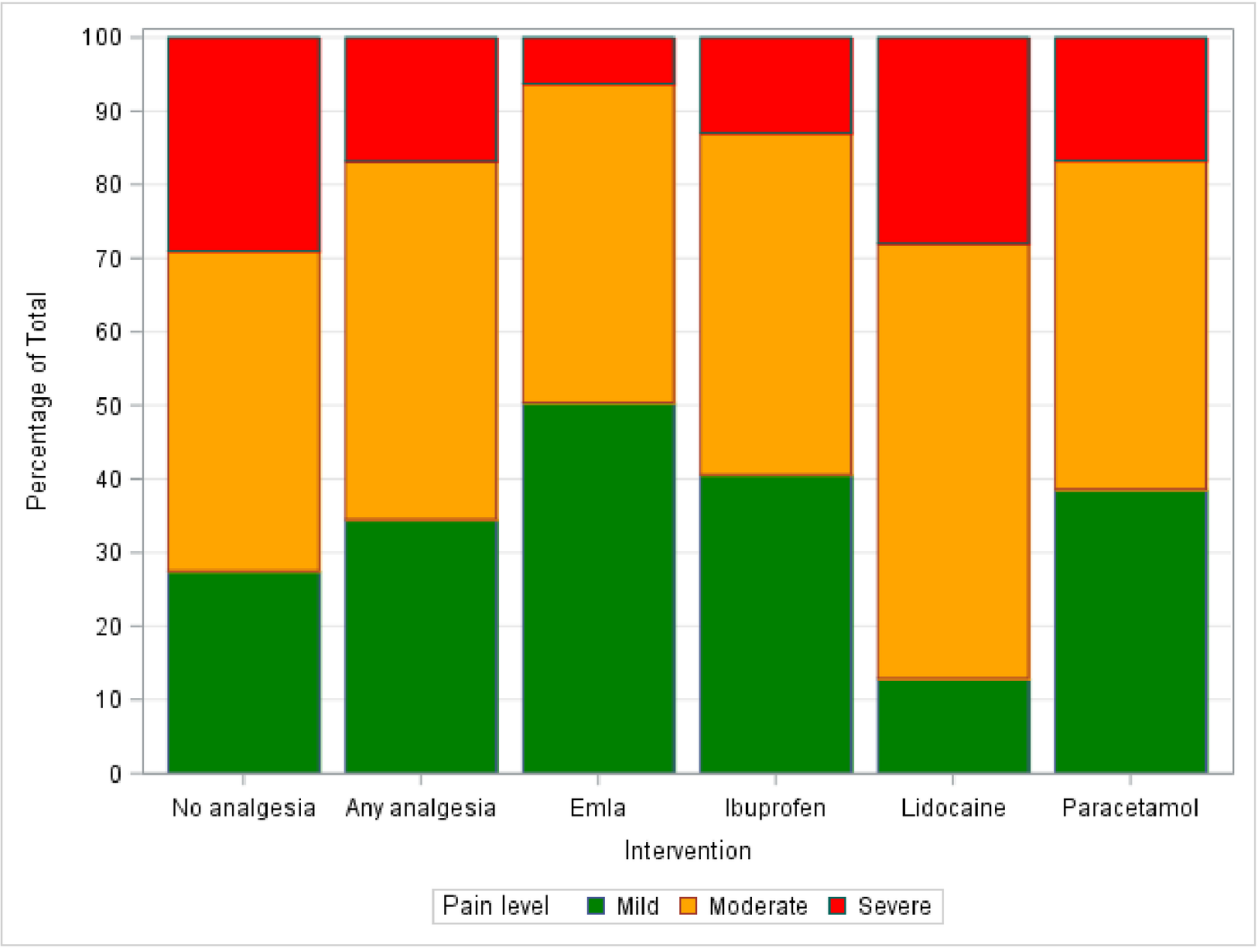
Different options of pain medication and pain level during removal.

Providing analgesia for device removal was dependent on the service provider and the availability of analgesia at the site. As a result, several (topical, oral) options were used. One fifth, 207 (21%), of participants did not receive any analgesia. Self-report of moderate pain did not vary much according to the type of analgesia given. A smaller proportion of participants who received any form of analgesia experienced severe pain compared to those who did not receive analgesia (16.6% vs. 29%, p = 0.0001) (Table 2). Paracetamol-codeine and ibuprofen had lower proportions of participants reporting severe pain (Table 2, Fig 2). Those that received EMLA and Oral Combination had the lowest self-report of severe pain during removal compared to those who received no analgesia (6.3% vs. 29%, p<0.0001).

**Table 2:** Pain comparison during PrePex device removal.

Drug Comparison	Moderate			Severe		
	Proportions		p-value	Proportions		p-value
Any Medication^#^ vs. No Analgesia	386/792 (48.7%)	90/207 (43.5%)	0.1774	133/792 (16.8%)	60/207 (29.0%)	0.0001
EMLA and Oral Combination vs. No Analgesia	97/224 (43.3%)	90/207 (43.5%)	0.9708	14/224 (6.3%)	60/207 (29.0%)	<0.0001
Ibuprofen vs. No Analgesia	32/69 (46.4%)	90/207 (43.5%)	0.6746	9/69 (13.0%)	60/207 (29.0%)	0.0081
Lidocaine vs. No Analgesia	137/232 (59.1%)	90/207 (43.5%)	0.0011	65/232 (28.0%)	60/207 (29.0%)	0.8224
Paracetamol-Codeine vs. No Analgesia	118/264 (44.7%)	90/207 (43.5%)	0.7915	44/264 (16.7%)	60/207 (29.0%)	0.0014
Ibuprofen vs. EMLA and Oral Combination	32/69 (46.4%)	97/224 (43.3%)	0.6530	9/69 (13.0%)	14/224 (6.3%)	0.067
Lidocaine vs. EMLA and Oral Combination	137/232 (59.1%)	97/224 (43.3%)	0.0008	65/232 (28.0%)	14/224 (6.3%)	<0.0001
Paracetamol-Codeine vs. EMLA and Oral Combination	118/264 (44.7%)	97/224 (43.3%)	0.7573	44/264 (16.7%)	14/224 (6.3%)	0.0004
Ibuprofen vs. Lidocaine	137/232 (59.1%)	32/69 (46.4%)	0.0625	65/232 (28.0%)	9/69 (13.0%)	0.0112
Ibuprofen vs. Paracetamol-Codeine	118/264 (44.7%)	32/69 (46.4%)	0.8028	44/264 (16.7%)	9/69 (13.0%)	0.4638
Lidocaine vs. Paracetamol-Codeine	137/232 (59.1%)	118/264 (44.7%)	0.0014	65/232 (28.0%)	44/264 (16.7%)	0.0023

#There were 3 participants who received other medication and are only included in the ‘Any Medication’ analysis. Only 999 participants had VAS scores

## Discussion

This active surveillance study assessing the PrePex medical male circumcision device conducted in six clinics across four provinces in South Africa, clearly shows that pain at device removal can be controlled using topical or oral medication.

In this operational study, fewer than two percent of the participants were ineligible for PrePex device placement for various reasons including genital abnormalities. The proportion of ineligible participants in our study is lower than previously reported in other studies where those ineligible range from 6%-9% [16, 17] with the most common reasons being genital abnormalities and HIV infection [18]. The lower prevalence of genital abnormalities in our study can be explained by some healthcare workers providing information for PrePex after medical examination and therefore inadvertently excluding potential participants with genital abnormalities from being assessed for PrePex MMC. Phimosis and tight foreskin in this study were the commonest observed abnormalities, similar to data reported previously [16].

In this study 2.5% of the participants experienced adverse events. Overall the WHO report on PrePex device evaluation summarizes an adverse event prevalence of 1.7% from eight studies across three countries [19]. However, other studies have found the AE rate to vary from 1–2.8% [8,19]. The rate of AEs in this active surveillance was similar both to a previous PrePex pilot study (2.7%) and to surgical circumcision [5–7, 13]. The observed rate of displacements, early removals and self-removals was 1.5% (15/1023) which is higher than the reported 0.4% in the WHO report and other studies [20]. Although displacement and self-removal are rare adverse events, putting measures in place to ensure that all PrePex devices are removed at the right time, and at the facility where the devices were placed according to the guidelines requires more resources.

A previous pilot study in South Africa reported that participants experienced minimal pain during device placement. However, device removal was associated with moderate to severe pain that abated within half an hour[13]. Other studies have also reported on moderate and severe pain experienced during device removal for example: in Zimbabwe 28% of participants experienced mild to moderate pain during removal [21], in Botswana 6% and 2% had moderate to severe pain respectively [22], and in Mozambique 38% reported severe or unbearable pain and 22% had moderate pain during removal [18]. Although the manufacturer did not recommend analgesia for placement or removal, our findings indicate that providing analgesia prior to PrePex device removal reduced the proportion of participants reporting severe pain compared to no analgesia. Additionally, this non-randomized prospective cohort study suggests that EMLA and Oral Combination is effective at reducing pain during PrePex device removal. Topical medication may be more effective as it has a faster onset of action [23]. Severe pain during PrePex device removal can be minimized by offering pain medication to all participants 30 minutes to an hour prior to removal of PrePex device. Counselling on the transient pain during device removal could possibly prepare participants in managing the pain during the procedure. Failure to address pain during device removal might affect the acceptability of the procedure.

## Limitations

This study had several limitations. Firstly, this was a non-randomized and un-blinded study conducted in public sector and/or donor funded clinics within existing MMC program. Secondly, sites were encouraged to provide the analgesia they had available in the clinic, and so a wide variety of regimens were included in the analysis. Although all sites received training on collecting data, there was no quality assurance program that standardized the capture of pain responses across sites which could have led to bias. Some follow-up data was collected telephonically which limited clinical observations.

## Conclusion

This study found that PrePex MMC device adverse events in multiple clinics active surveillance field study are comparable to surgical circumcision. Our data strongly suggests that topical analgesia should be offered to all PrePex MMC device participants at least 30 minutes prior to removal to reduce the severe pain experienced during device removal. As the removal of the device is standardized, additional research to assess cost effective types and doses of analgesia is required. If PrePex is to be incorporated into national MMC programs these factors need to be considered and counselling interventions intensified in order to reduce pain associated with PrePex circumcision.

## Supporting information

S1 Prepex Surveillance Questionnaires (PDF)
