# Supplementary material for: PrePex circumcision surveillance: Adverse events and analgesia for device removal
Source: PLoS One. 2018 Mar 26;13(3):e0194271. doi: 10.1371/journal.pone.0194271 (PMC5868790; doi:10.1371/journal.pone.0194271)
Supplement: S1 Prepex Surveillance Questionnaires — (PDF) [file pone.0194271.s001.pdf]

BASELINE AND PLACEMENT

Version 4.1

June 2014

Participant's Initials:
Participant's SIN:
Date of Visit:

**Date of Birth** (dd/mm/yyyy):

GENERAL HOUSEHOLD AND EMPOLYMENT INFORMATION

Dwelling you are living in: ___ ___

1= Brick/concrete House
2= Apartment
3= Traditional hut/dwelling
4= Garage
5= 1 room
6= Shack
7 =Homeless
9= Other

If other specify: ____

Access to piped water: ___ ___

1= Piped water inside the dwelling

2= Piped water inside the yard

3= Piped water on a community stand <100m of household

4= Piped water within 100m of household but not inaccessible

5= Piped water inaccessible

Source of water: ___ ___

1= Government piped water

2= Borehole

3= Spring

4= Dam/pool/stagnant water

5= River or stream

9= Other

If other specify: ____

Participant SIN: ___________________

**Residential Address:**				
**Description of Area of residence:** ___ ___<br>**1=Farm**<br>**2=Location/township**<br>**3=Suburb**<br>**4= Town/City**<br>**5=Village**				
**Contact number:**				
**Employed currently?**	**Yes**		**No**	
**If Yes, are you a miner?**	**Yes**		**No**	
**If miner or doing hard physical work in the mining industry:** ___ ___<br>**1 = Underground** **2 = Above ground**				

GENERAL MEDICAL HISTORY

HIV Serostatus

	**Positive**	**Negative**
Participant's HIV Serostatus	☐	☐
If "Negative," document the date of test:<br><br>*Counselor's name:*		
If "Positive, CD4 count __ __ __ __cells/mm3<br><br>*Date taken (dd/mm/yyyy):*<br>*(must be within two months of circumcision and above 350 cells.)*		

Based on general examination and medical history provided by the participant, is he suitable for Prepex Medical Male Circumcision? YES ☐ NO ☐
**If NO, give reasons:**

**If the individual has diabetes, he is ineligible for the study.*

PHYSICAL EXAMINATION, VITAL SIGNS & MEDICATION

Body System	Normal	Abnormal	Comments
Genital	☐	☐	

Current Medication

	Yes	No
Is the participant currently taking medication?	☐	☐

Participant SIN: ____________________

If yes, record medication below						
**Medication Name (generic)**	**Medication Dosage**			**Medication Course**		**Ongoing**
	**Units**	**Frequency**	**Route**	**Start**	**Stop**	
1.	☐☐	☐☐	☐☐	Date:	Date:	☐
2.	☐☐	☐☐	☐☐	Date:	Date:	☐
3.	☐☐	☐☐	☐☐	Date:	Date:	☐

Units: 01. Gram (g); **02.** Milliliter (ml); **03.** Milliequivalent (MEQ); **04.** Milligram (mg); **05.** Drops; **06.** International Units (IU); **07.** Unknown
Frequency: 01. Once a day; **02.** Every night; **03.** Twice a day; **04.** Three times a day; **05.** Four times a day; **06.** Five times a day; **07.** Once a week; **08.** Every 6 hours; **09.** Every 8 hours; **10.** Every 12 hours; **11.** As needed; **12.** Unknown
Route: 01. Intravenous; **02**. Oral; **03.** Rectal; **04.** Intramuscular; **05.** Intradermal; **06.** Inhalation; **08.** Subcutaneous; **09.** Sublingual; **10.** Topical; **11.** Unknown

	**Yes**	**No**
Following medical examination is the participant suitable for PrePex Medical Male Circumcision	☐	☐
If not, why?		

Device Placement

Did this person refuse application after signing informed consent? YES ☐ NO ☐

Date of Device Application (dd/md/yyyy): ____________________

If the date is different from the screening visit, record the participant's vital signs and genital condition.

Vital Signs

Blood Pressure	/ mm Hg		Pulse	b/m
Genital condition	Normal	☐	Abnormal	☐

Start Time (Cleaning Patient Genital)	
Placement Completed (Patient Getting off the bed)	

**Operator**	
**Assistant**	

Participant SIN: ____________________

Visual Analog Scale

Evaluation of pain level per VAS during placement	☐☐

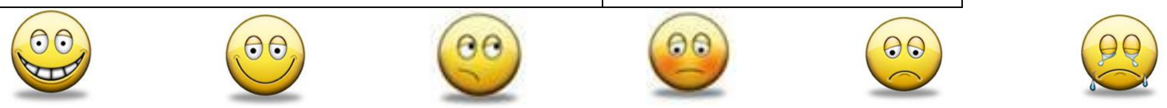

0	2	4	6	8	10
Very happy, no hurt	Hurts just a little bit	Hurts a little more	Hurts even more	Hurts a whole lot	Hurts as much as you can imagine

Prepex Device

Measured PrePex Size	A ☐	B ☐	C ☐	D ☐	E ☐	Other ☐	No Size ☐
If the device application is not the same day as the screening, has there been a change in the genitals (compare to screening visit report)?				Yes ☐	No ☐		
Was the device applied according to its Instructions For Use?<br>If "No," explain in detail:				Yes ☐	No ☐		
Provider evaluation of application/placement from 1 to 10 (1 being worst):				☐☐			

Prepex Device Details

**SIZE**		**OR ATTACH PREPEX LABEL**
**LOT. NO**		
**MAN. Date**		
**EXP. Date**		

ADVERSE EVENTS DURING PLACEMENT

Were there any device related events or AE that occurred in this visit? Yes ☐ No ☐

If "Yes", please record below.

Onset/Start

Date of AE: __ __/__ __/201__

Time of AE: __ __: __ __

Participant SIN: ____________________

Date/time observed (if different) ______________________

Status of AE: New ☐ Ongoing ☐

Diagnosis: Primary ☐ Secondary ☐

Tick the relevant AE and classification

Pain ☐	Mild ☐<br>Moderate ☐<br>Severe (complete SAE form) ☐
Bleeding ☐	Mild ☐<br>Moderate ☐<br>Severe (complete SAE form) ☐
Analgesia/Anesthesia-Related ☐	Mild ☐<br>Moderate ☐<br>Severe (complete SAE form) ☐
Damage to penis ☐	Mild ☐<br>Moderate ☐<br>Severe (complete SAE form) ☐
Other: *Please specify*<br>______________________________________	Mild ☐<br>Moderate ☐<br>Severe (complete SAE form) ☐

Describe the AE

____

____

____

Action:

☐ None ☐ Surgical ☐ Non-Surgical ☐ Pharmaceutical

☐ Study termination ☐ Other: __________________________________

DEVICE EVENTS

Was there any difficult during placement? Yes ☐ No ☐

If 'Yes' Describe difficulty during placement

____
____
____

Were there any other events that occurred during the placement that you would like to record? (e.g. crying, shouting, needing excessive reassurance prior to or during procedure, wanting to stop procedure e.t.c.)

Yes ☐ No ☐

If 'Yes' describe

____
____
____
____

I have checked all data introduced to this form until this point and verified that they are complete, correct and reconcilable with the original documentation.

Name:	Position:
Signature:	Date:

Visit #2: DEVICE AND FORESKIN REMOVAL<br>Version 1.5<br>July 2014
**Day 7**

Participant's Initials:
Participant's SIN:
Date of visit:

Work Leave

Did the participant take day(s) off from work following the circumcision?

Yes ☐ No ☐ NA ☐

If "Yes", number of days:

1 ☐ 2 ☐ 3 ☐ 4 ☐ 5 ☐

**Pain – Before removal**

Visual Analog Scale – Before Removal

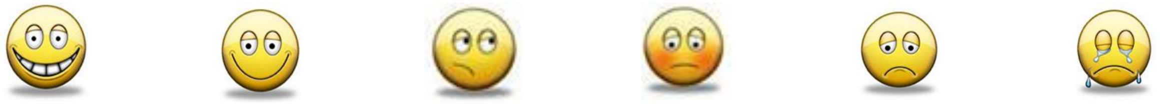

0	2	4	6	8	10
**Very happy, no hurt**	**Hurts just a little bit**	**Hurts a little more**	**Hurts even more**	**Hurts a whole lot**	**Hurts as much as you can imagine**

Write the number associated with the face that best describes pain level in the blocks provided

1. Evaluation of pain level per VAS <u>before</u> removal procedure: ☐ ☐

1.1. Has the participant experienced anything else that they did not like related to the circumcision apart from pain or discomfort?

Yes ☐ No ☐

1.2. *If "Yes",* what was it they experienced?

____

____

____

____

____

Participant SIN: ____________________

Current Medication

	Yes	No
Has there been any change to participant's medication since device placement?	☐	☐
If yes, record medication below		

Medication Name (generic)	Medication Dosage			Medication Course		Ongoing
	Units	Frequency	Route	Start	Stop	
1.	☐☐	☐☐	☐☐	Date:	Date:	☐
2.	☐☐	☐☐	☐☐	Date:	Date:	☐
3.	☐☐	☐☐	☐☐	Date:	Date:	☐

Units: 01. Gram (g); **02.** Milliliter (ml); **03.** Milliequivalent (MEQ); **04.** Milligram (mg); **05.** Drops; **06.** International Units (IU); **07.** Unknown
Frequency: 01. Once a day; **02.** Every night; **03.** Twice a day; **04.** Three times a day; **05.** Four times a day; **06.** Five times a day; **07.** Once a week; **08.** Every 6 hours; **09.** Every 8 hours; **10.** Every 12 hours; **11.** As needed; **12.** Unknown
Route: 01. Intravenous; **02**. Oral; **03.** Rectal; **04.** Intramuscular; **05.** Intradermal; **06.** Inhalation; **08.** Subcutaneous; **09.** Sublingual; **10.** Topical; **11.** Unknown

Removal Procedure

Start Time (Cleaning Patient Genital)	
Removal Completed (Patient Getting off the bed)	

**Operator Name**	
**Assistant Name**	

Pain –During removal of PrePex

Visual Analog Scale – Pain During Removal of PrePex

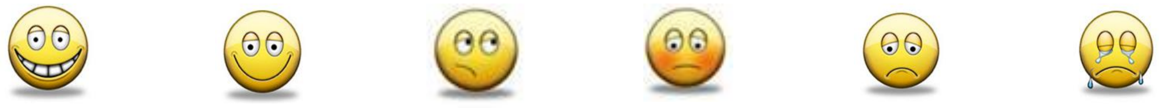

0	2	4	6	8	10
Very happy, no hurt	Hurts just a little bit	Hurts a little more	Hurts even more	Hurts a whole lot	Hurts as much as you can imagine

Write the number associated with the face that best describes pain level in the blocks provided

2. Evaluation of pain level per VAS <u>during</u> removal procedure: ☐☐

**Removal Particulars**		
5.1 Was preventive pain medication administered to the patient in the <u>hour prior to the removal</u> of PrePex, specifically to prevent pain of removal?<br>*If "Yes",* What medication?<br>Paracetamol alone ☐<br>Codeine alone ☐<br>Paracetamil with codeine combination tablets ☐<br>Ibuprofen alone ☐<br>Emla cream ☐<br>Other ☐<br>If other, specify:_______________________________	Yes ☐	No ☐
5.2 Was Lidocaine spray used<br>*If "Yes"*<br>Evaluation of pain level per VAS after removing the elastic ring<br>☐☐<br>Evaluation of pain level per VAS after removing the inner ring<br>☐☐<br>Evaluation of pain per VAS 15 minutes after removal<br>☐☐	Yes ☐	No ☐
5.3 Was any medication or anesthesia required <u>during removal</u>?<br>*If "Yes",* What?____________________________	Yes ☐	No ☐
5.4 Was a spatula used to remove the inner ring?	Yes ☐	No ☐
5.5 Was a finger used to remove the inner ring?	Yes ☐	No ☐
5.6 Were there any device related events or AE that occurred during the removal?<br>*If "Yes", please record on the device related events form and adverse events form, if needed.*	Yes ☐	No ☐

5.7	How would you rate the removal procedure from 1 to 10 (with 1 being worst): ☐ ☐		

ADVERSE EVENT

6. Has the participant experienced an AE since ther last visit or at this visit?

Yes ☐ No ☐

If 'Yes' complete below

6.1. Onset/Start

Date of AE: __ __/__ __/201__

Time of AE: __ __:__ __

Date/time observed (if different) ______________________

6.2. Status of AE: New ☐ Ongoing ☐
6.3. Diagnosis: Primary ☐ Secondary ☐

6.4. Tick the relevant AE and classification

Pain ☐	Mild ☐<br>Moderate ☐<br>Severe (complete SAE form) ☐
Bleeding ☐	Mild ☐<br>Moderate ☐<br>Severe (complete SAE form) ☐
Amount of blood: PLEASE RECORD ANY BLEEDING	Minimal ☐<br>At least 5ml ☐<br>10-40ml ☐<br>>50ml ☐
Intervention: This means did the person removing PrePEx or anyone else provided an intervention or treatment that they would not normally have done for a PrePex patient	No intervention/treatment required ☐<br>Required local pressure to stop the bleeding ☐<br>Other

Participant SIN: ____________________

	______________________________________<br>______________________________________

Infection	☐	Mild<br>Moderate<br>Severe (complete SAE form)	☐<br>☐<br>☐
Hematoma	☐	Mild<br>Moderate<br>Severe (complete SAE form)	☐<br>☐<br>☐
Problems Voiding	☐	Mild<br>Moderate<br>Severe (complete SAE form)	☐<br>☐<br>☐
Damage to Penis	☐	Mild<br>Moderate<br>Severe (complete SAE form)	☐<br>☐<br>☐
Wound Dehiscence	☐	Mild<br>Moderate<br>Severe (complete SAE form)	☐<br>☐<br>☐
Other:	☐	Mild<br>Moderate<br>Severe (complete SAE form)	☐<br>☐<br>☐

7. Describe the AE

____

____

____

8. Action:

☐ None ☐ Surgical ☐ Non-Surgical ☐ Pharmaceutical

☐ Study termination ☐ Other: ___________________________________

DEVICE EVENTS

9. Was there any device:

Displacements ☐ Early removal ☐

Detachment ☐ Self removal ☐

9.1. If 'Yes' Describe condition resulting in either of the above mentioned

____
____
____
____

10. Was there difficulty during device removal?

Yes ☐ No ☐

10.1. If 'Yes', describe the difficulty experienced during removal

____
____
____

Name:	Position:
Signature:	Date:

PARTICIPANT REVIEW FORM
To be used at any contact after Prepex Application Except Day of Removal
**Version 1.5**
**July 2014**

Participant's Initials:
Participant's SIN:
Date:

**Scheduled Visit**		**Unscheduled Visit**	
**Visit Occurred while device ON**		**Visit Occurred while Device OFF**	
**Day of Visit Day: Visit 21** ☐ **Visit 49** ☐ **Other:**			
**Telephonic** ☐ **or Clinical Visit** ☐ **or Home Visit** ☐			
**Conform spoken to patient** ☐ **or Spoken to a relative/friend** ☐			

1.1. Has the participant experienced anything else that they did not like related to the circumcision apart from pain or discomfort?

Yes ☐ No ☐

1.2. *If "Yes"*, what was it they experienced?

____
____
____

Current Medication

	**Yes**	**No**
Has there been any change to the participant's medication since last visit?	☐	☐
If yes, record medication below		

**Medication Name (generic)**	**Medication Dosage**			**Medication Course**		**Ongoing**
	**Units**	**Frequency**	**Route**	**Start**	**Stop**	
1.	☐☐	☐☐	☐☐	Date:	Date:	☐
2.	☐☐	☐☐	☐☐	Date:	Date:	☐
3.	☐☐	☐☐	☐☐	Date:	Date:	☐

Participant SIN: ________________________

Units: 01. Gram (g); **02.** Milliliter (ml); **03.** Milliequivalent (MEQ); **04.** Milligram (mg); **05.** Drops; **06.** International Units (IU); **07.** Unknown

Frequency: 01. Once a day; **02.** Every night; **03.** Twice a day; **04.** Three times a day; **05.** Four times a day; **06.** Five times a day; **07.** Once a week; **08.** Every 6 hours; **09.** Every 8 hours; **10.** Every 12 hours; **11.** As needed; **12.** Unknown
Route: 01. Intravenous; **02**. Oral; **03.** Rectal; **04.** Intramuscular; **05.** Intradermal; **06.** Inhalation; **08.** Subcutaneous; **09.** Sublingual; **10.** Topical; **11.** Unknown

Wound Description

____

____

____

Device related event or Adverse Event

1. Has the participant experienced any AE since the PrePex device was applied?

Yes ☐ No ☐

If 'Yes' complete below

1.1. Onset/Start

Date of AE: __ __/__ __/201__

Time of AE: __ __: __ __

Date/time observed (if different) ____________________

1.2. Status of AE: New ☐ Ongoing ☐
1.3. Diagnosis: Primary ☐ Secondary ☐

1.4. Tick the relevant AE and classification

Pain	☐	Mild<br>Moderate<br>Severe (complete SAE form)	☐<br>☐<br>☐
Bleeding	☐	Mild	☐

	Moderate ☐<br>Severe (complete SAE form) ☐
Amount of blood:	Minimal ☐<br>At least 5ml ☐<br>10-40ml ☐<br>>50ml ☐
Intervention:	No intervention/treatment required<br>☐<br>Required local pressure to stop the bleeding<br>☐<br>Other:<br>________________________________<br>________________________________
Infection ☐	Mild ☐<br>Moderate ☐<br>Severe (complete SAE form) ☐

Hematoma ☐	Mild ☐<br>Moderate ☐<br>Severe (complete SAE form) ☐
Problems Voiding ☐	Mild ☐<br>Moderate ☐<br>Severe (complete SAE form) ☐
Damage to Penis ☐	Mild ☐<br>Moderate ☐<br>Severe (complete SAE form) ☐
Wound Dehiscence ☐	Mild ☐<br>Moderate ☐<br>Severe (complete SAE form) ☐

Excessive skin removed ☐	Mild ☐<br>Moderate ☐<br>Severe (complete SAE form) ☐
Insufficient skin removed ☐	Mild ☐<br>Moderate ☐<br>Severe (complete SAE form) ☐
Torsion ☐	Mild ☐<br>Moderate ☐<br>Severe (complete SAE form) ☐
Other: *Please specify* ☐<br>___________________________________	Mild ☐<br>Moderate ☐<br>Severe (complete SAE form) ☐

2. If the participant experienced any of the below then tick and describe.

Delayed Wound Healing Yes ☐ No ☐<br>If 'Yes', Describe:<br>____<br>____<br>____
Scarring/disfigurement Yes ☐ No ☐<br>If 'Yes', Describe:<br>____<br>____<br>____
Erectile Dysfunction Yes ☐ No ☐<br>If 'Yes' Describe: -<br>____<br>____<br>____<br>Frequency: ☐☐<br>Timing with regard to circumcision: ______________________
Psychosocial behavior Yes ☐ No ☐<br>If 'Yes' Describe:<br>____<br>____<br>____
Timing or onset:

Participant SIN: ________________________

3. Describe the AE

____
____
____

4. Action:

☐ None ☐ Surgical ☐ Non-Surgical ☐ Pharmaceutical

☐ Study termination ☐ Other: ________________________________

Appearance after circumcision

5. Are you satisfied with the appearance of your penis post circumcision?
   Yes ☐ No ☐

If "No" then explain why not and what you expected to see

____
____
____

6. Is the wound healed completely?
   Yes ☐ No ☐
7. Is the participant terminated from the study according to the protocol?

   7.1 Yes ☐ No ☐
   7.1.1 If 'No' – Date of Next Visit:____________________________

   7.2 Reason for termination if participant terminated NOT according to protocol

   Loss to follow up ☐ Moved out of area ☐
   Unable to contact ☐ Death ☐
   Other: ____

Name:	Position:
Signature:	Date:

Participant's Initials:	
Participant's SIN:	Page Number ___

Concomitant Medication Form

USE ONE FORM FOR THE ENTIRE STUDY
UPDATE THIS FORM AT EACH VISIT
IF NECESSARY GO TO A SECOND OR THIRD PAGE

Has the participant taken concomitant medication (including pain killers) since the last visit?
Y N
If "No", do not enter information on this form.
If "Yes", please complete below.

Medication Name (generic)	Medication Dosage			Medication Course		Ongoing
	Units	Frequency	Route	Start	Stop	
1.	☐☐	☐☐	☐☐	Date:<br>Time:	Date:<br>Time:	☐
2.	☐☐	☐☐	☐☐	Date:<br>Time:	Date:<br>Time:	☐
3.	☐☐	☐☐	☐☐	Date:<br>Time:	Date:<br>Time:	☐
4.	☐☐	☐☐	☐☐	Date:<br>Time:	Date:<br>Time:	☐
5.	☐☐	☐☐	☐☐	Date:<br>Time:	Date:<br>Time:	☐
6.	☐☐	☐☐	☐☐	Date:<br>Time:	Date:<br>Time:	☐
7.	☐☐	☐☐	☐☐	Date:<br>Time:	Date:<br>Time:	☐

Units: 01. Gram (g); **02.** Milliliter (ml); **03.** Milliequivalent (MEQ); **04.** Milligram (mg); **05.** Drops; **06.** International Units (IU); **07.** Unknown

Frequency: 01. Once a day; **02.** Every night; **03.** Twice a day; **04.** Three times a day; **05.** Four times a day; **06.** Five times a day; **07.** Once a week; **08.** Every 6 hours; **09.** Every 8 hours; **10.** Every 12 hours; **11.** As needed; **12.** Unknown

Route: 01. Intravenous; **02**. Oral; **03.** Rectal; **04.** Intramuscular; **05.** Intradermal; **06.** Inhalation; **08.** Subcutaneous; **09.** Sublingual; **10.** Topical; **11.** Unknown

Name:	Position:
Signature:	Date:

Severe Adverse Events (SAE) Form

Participant's Initials:
Participant's SIN:

Is there a photographer present to take a photo of the SAE

Yes ☐ **No** ☐

Do not continue until a photographer is present

Onset/Start

Date of SAE: __ __/__ __/201__

Time of SAE: __ __:__ __

Date/time observed (if different) ______________________

Number of SAE for this participant? ☐ ☐

Status

New ☐

Ongoing ☐

Description

Please describe SAE in block letters

____

____

____

____

Criteria

Chose criteria for SAE

☐ Death

☐ Permanent disability

☐ Life threatening

☐ Hospitalization or prolongation of inpatient hospitalization

☐ Required intervention to prevent one of the above

☐ Other:

____

____

Outcome

☐ Resolved

☐ Resolved with sequel

☐ Persistent

☐ Unchanged

☐ Unknown

End/Termination

Date of SAE: __ __/__ __/201__

Time of SAE: __ __:__ __

Please add any additional comments related to this AE. Please write in block letters and sign and date your comments.

____

____

____

____

____

Does Human Research Ethics Committee need to be immediately notified of AE? Y N

If Y please draft notification and send notification now.

I have checked all data introduced to this form until this point and verified that they are complete, correct and reconcilable with the original documentation.

Name:	Position:
Signature:	Date:
